# Movement synchrony among dance performers predicts brain synchrony among dance spectators

**DOI:** 10.1038/s41598-024-73438-0

**Published:** 2024-09-27

**Authors:** Guido Orgs, Staci Vicary, Matthias Sperling, Daniel C. Richardson, Adrian L. Williams

**Affiliations:** 1 Institute of Cognitive Neuroscience, University College London, London, UK; 2 School of Psychological Sciences, University of Newcastle, Sydney, Australia; 3 Independent Artist and Choreographer, London, UK; 4 Department of Experimental Psychology, University College London, London, UK; 5 Division of Psychology, Department of Life Sciences, Brunel University London, Uxbridge, UK

**Keywords:** Dance, Inter-subject correlations, Synchrony, Joint action, Neuroaesthetics, fMRI, Action observation, Cognitive neuroscience, Human behaviour, Perception

## Abstract

Performing dance is an intrinsically social art form where at least one person moves while another person watches. Dancing in groups promotes social bonding, but how does group dance affect the people watching? A group of dancers and dance novices watched a 30 min dance video individually in an fMRI scanner. In a follow-up behavioural study, the same people watched the video again and provided continuous enjoyment ratings. Firstly, we computed cross-recurrence of continuous enjoyment ratings and inter-subject correlations (ISCs) in fMRI separately for both groups, and with the choreographer of the dance work. At both behavioural and neural levels, dancers responded more similarly to each other than novices. ISCs among dancers extended beyond brain areas involved in audio-visual integration and sensory areas of human movement perception into motor areas, suggesting greater sensorimotor familiarity with the observed dance movements in the expert group. Secondly, we show that dancers’ brain activations and continuous ratings are more similar to the choreographer’s ratings in keeping with sharing an aesthetic and artistic perspective when viewing the dance. Thirdly, we show that movement synchrony among performers is the best predictor of brain synchrony among both expert and novice spectators. This is consistent with the idea that changes in emergent movement synchrony are a key aesthetic feature of performing dance. Finally, ISCs across perceptual and motor brain areas were primarily driven by movement acceleration and synchrony, whereas ISCs in orbital and pre-frontal brain areas were overall weaker and better explained by the continuous enjoyment ratings of each group. Our findings provide strong evidence that the aesthetic appreciation of dance involves a common experience between dance spectators and the choreographer. Moreover, the similarity of brain activations and of enjoyment increases with shared knowledge of - and practice in - the artform that is being experienced, in this case contemporary performing dance.

## Introduction

Performing dance and music are fundamentally social art forms, often performed and experienced in groups^1–4^. Whether in theatres, concert halls, cinemas or galleries, experiencing art together rather than alone can greatly enhance our engagement with an artwork^5^.

If collective aesthetic experiences involves a common experience between at least two people, cognitive neuroscience suggests that they involve a form of mutual entrainment between spectators or listeners^6^. In live performance contexts, it has been shown that spectators synchronise their heartrate and breathing rate when watching dance or a fire ritual^7,8^ and synchronise their head movements when watching a rock concert^9^. In fMRI, brain synchrony between people is often measured by computing inter-subject correlations (ISCs) while people watch naturalistic stimuli such as film. Brain activity between multiple movie watchers predicts better memory for movie content^10,11^ and affective responses to TV series and ads^12,13^. Brain synchrony in these studies reflects a combination of both exogenous stimulus features and endogenous, more idiosyncratic processing of these features^12,14,15^. In particular, sensory areas in posterior regions of the brain are more closely tied to stimulus features, whereas brain synchrony in the orbital and medial frontal cortices is more closely tied to subjective experience, including aesthetic judgement^15,16^.

Brain synchrony has been argued to reflect shared emotions among people watching movies, dance or listening to stories. In one study, when listening to emotional stories, brain synchrony was correlated with heart rate measures as well as valence and arousal ratings after scanning. Negative shifts in valence were associated with greater phase synchrony in ACC and S1, and midline brain regions typically associated with self-referential processing. Positive shifts in valence were associated with greater phase synchrony in medial prefrontal cortices. In contrast increased arousal was related to greater brain synchrony in medial prefrontal and auditory areas; a decrease in arousal was associated with greater brain synchrony in visual cortices, the middle cingulum, the precuneus and supplementary motor areas^6^. More recent work has also shown that brain synchrony correlates with the rhetorical quality of political speeches^17^. Interestingly, ISCs during movie viewing are greater if spectators are instructed to take the same psychological perspective and the pattern of eye movements does not predict differences in ISCs^18^.

Much research using ISCs is conducted with narrative stimuli (audiobook, TV episodes and film) in which peak brain synchronisation typically occurs at salient turning points in the plot or during highly emotionally laden moments. Yet, research in joint action perception suggests that mere observation of other people moving together is rewarding in itself, in the absence of any storyline^19^. In dance, the synchrony of group dancing is linked to group affiliation^20^ and predicts how much audiences enjoy a live dance performance^21^. In the latter study, synchrony of movement acceleration was measured continuously among a group of ten dancers while they were performing a 30-min choreography. Additionally, continuous heart rate and enjoyment ratings were collected from spectators. Synchrony predicted continuous enjoyment ratings and heart rate, but only if spectators formed a consistently positive or negative aesthetic evaluation of the dance performance.

Only very few neuroimaging studies have applied ISCs to study brain synchrony while watching dance. One study compared brain synchrony among dance naïve spectators during the observation of edited and unedited videos of a single ballet dancer^22^. Viewing the dance videos—which contained both music and movement—was associated with shared activations in brain areas related to audio-visual integration, bilaterally in the posterior superior temporal gyri (STG) and with activations in the human action observation network, such as motor and premotor cortices and the superior parietal lobe. Brain synchrony in STG was stronger for unedited as compared to edited videos, perhaps indicating better alignment between auditory visual information in the unedited condition, see also^23^. One study combined a qualitative assessment of the spectators’ live experience of watching dance with a follow-up fMRI experiment in which a video of the same performance was watched by a different group of spectators with different musical soundtracks^24^. Spectators in the scanner watched two versions of the choreography: in one condition, the choreography was performed to classical music, in the second condition it was performed without music so that only the breathing and footfalls were audible. Brain synchrony among spectators was most pronounced in superior temporal and middle occipital cortices, but also included frontal motor areas as well the superior parietal lobule. Interestingly, brain synchrony in the breathing only condition was stronger in the superior temporal gyrus and involved the right postcentral gyrus. The authors argue that greater synchrony in the STG reflects the perfect simultaneity of auditory and visual input in the breathing only condition. The authors argue that these activations in somatosensory cortex might reflect ‘kinaesthetic empathy’ i.e. engagement of somatosensory areas in participants, that was triggered by the breathing sounds and footfalls of the performers. Together these findings suggest that watching dance does not only synchronize brain areas involved in audio-visual integration of movement and sound, but also involves the human action observation network^25^. However, none of these studies on dance perception showed activation in prefrontal brain areas related to reward processing, although these areas have been reported more consistently for the aesthetic appreciation of images, paintings^16,26,27^ and music^28,29^. Similarly, it is not known whether greater brain synchrony can be linked to specific features of the movement, for example movement complexity^30^ or synchrony among performers^21,31^.

Dance observation depends on dance expertise which modulates brain activations in visual, parietal and motor areas of the action observation network^31–34^. Learning to perform a movement increases the aesthetic appeal of these movements, and correlates with greater activation in the left superior temporal sulcus for these movements^35^. It is however an open question as to how dance training impacts on brain synchrony when watching dance. One behavioural study on the aesthetics of mathematical equations suggests that expertise produces greater agreement, based on shared formal training and knowledge in maths^36^, but is such greater agreement measurable at the neural level? Arguably, contemporary dancers might not only share more knowledge with *each other* about contemporary dance, but also with *a maker /choreographer* of a contemporary dance work as they are more likely to understand the choreographic principles, dance practices and cultural context within which an artwork was made^37,38^. In this way, greater brain synchrony between experienced dancers and choreographers watching a specific dance work might reflect a shared artistic and aesthetic perspective^18^.

In the present study, we compare ISCs between dancers and dance novices while watching the recording of a live dance performance and use kinematic measures collected during the live performance to predict ISCs among both groups. We propose that the size of ISCs will covary with both dance experience and salient aesthetic features of the observed performance, in this case synchronous movement among a group of dance performers^20,21^. We will test three hypotheses at behavioural and neural levels: Firstly, we predict greater overall ISCs and behavioural agreement in the dance experienced group relative to the dance novice group (H1). Secondly, we predict that both continuous ratings of enjoyment and brain activations will be more similar between the choreographer and the dancers, than between the choreographer and the novices (H2). Thirdly, we predict that ISCs in dancer and novice spectators are best explained by movement synchrony among performers and that stimulus features should predict ISCs across posterior sensorimotor areas of the brain, whereas enjoyment ratings should better explain ISCs across prefrontal areas related to reward and self-referential processing (H3).

## Methods

### Participants

Existing studies that use ISCs in the context of dance viewing^22,23^ tested between 12 and 16 participants, albeit using stimuli with a much shorter video duration than in our study (< 7 min vs. 30 min), we therefore aimed for a total sample size of 30 participants^39^. Twenty-eight people took part in the study, including the choreographer of the dance performance (MS, age = 40, 30 years of dance experience, left-handed). Two fMRI data sets were lost, one novice participant due to technical error, and one dancer based on adverse experience in the scanner, resulting in 11 novice 14 dancer fMRI datasets. Dance novices (Mean age = 31 years, SD = 7 years), had no formal dance training and less than 5 years of casual involvement with dance (for example taking community classes or dance for fitness). Professional dancers (M age = 28 years, SD = 7 years), had between 9 and 33 years of professional dance experience (mean experience = 17 years). All participants were right handed, except for the choreographer and one dancer who reported left handedness as assessed by the Flinders handedness survey^40^. Two participants were unavailable for the follow up session and five follow up sessions resulted in incomplete continuous enjoyment data (three sessions due to technical error, two due to user error). The final sample for continuous enjoyment data is N = 19, with 6 sessions from novices and 13 sessions recorded from dancers. All participants provided informed consent, and all data collection and data analyses were carried out in accordance with relevant guidelines and regulations. The research was approved by the ethics committee at Brunel University London.

## Materials

### Dance video

**Fig. 1:**
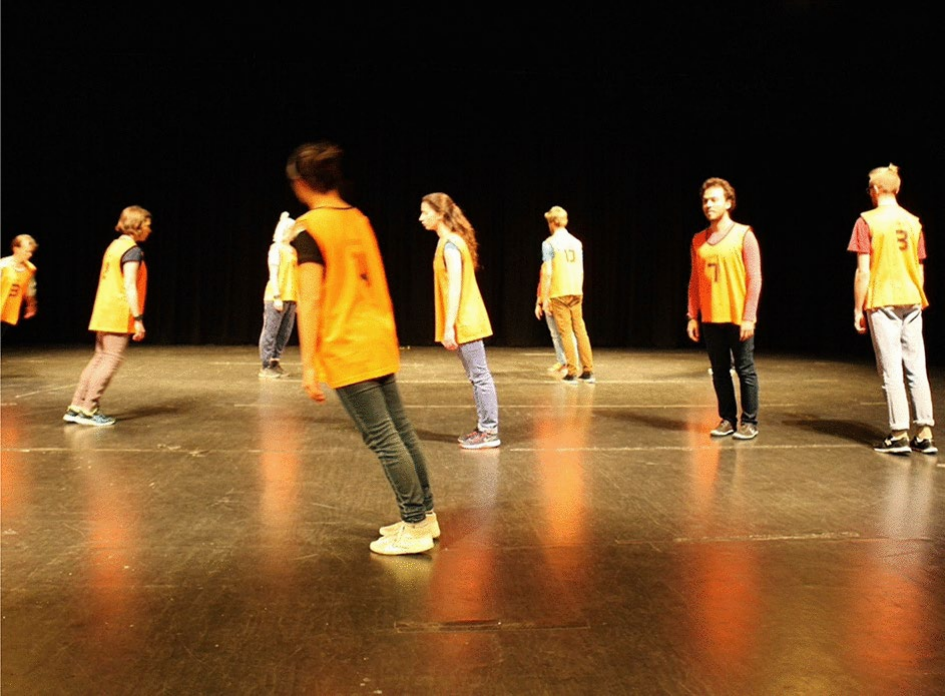
Snapshot of *Group Study* by Matthias Sperling (2015). Movement acceleration from all ten performers was recorded using Empatica E4 wrist sensors. Image by Matthias Sperling.

Participants viewed a recorded version of the choreography *Group Study* by Matthias Sperling. This work was developed in the context of research on the perception of movement synchrony in dance. In *Group Study*, 10 professional dancers perform a task-based choreography that consist of mainly pedestrian movements such as walking, running and arm swinging. Over the course of the performance, movement synchrony systematically varies among performers, with moments of near perfect synchrony (i.e. all dancers stop moving at the same time) and moments where dancers move completely independently. In *Group Study*, synchrony and asynchrony are emergent properties of the live interactions between performers. In contrast with conventional ideas of synchrony in dance, this approach relies neither on the repetition of pre-planned movement sequences, nor external signals (such as music) to establish group co-ordination (Fig. 1). Accordingly, *Group Study* involves vocalizations and the sound of the performers moving, but no music. The video was filmed frontally in a single shot without editing and lasted 33 min and 54 s, the video used in this study is available to view at https://research.gold.ac.uk/id/eprint/34238/. For detailed information on the performance and the choreographic score, see^21^.

#### Quantification of the performers’ movements

During the dance performance, we recorded the movements of all 10 performers using wrist accelerometers (Empatica E4 wrist sensors^41^) and computed measures of overall acceleration and synchrony using cross-recurrence analysis. In addition, motion and sound energy were also extracted from the dance video.

We calculated an overall measure of how much performers moved by taking the square root of the sum of the squared x, y and z -axis values of the acceleration data, leaving a single time series vector for each of the 10 performers. Acceleration was then averaged across performers within 2 s time windows. To compute movement synchrony, we applied cross-recurrence quantification analysis (CRQA) to the non-windowed acceleration vectors to obtain a continuous measure of synchrony among performers across time using the CRQA package implemented in R. Cross recurrence was calculated with an embedding dimension of 1, a radius of 10 and a delay of 1^42^. The recurrence rate was calculated for every possible pair of performers (N = 90 pairs), within a + /− 2 s lag window, for more detail, see^21^.

Finally, we extracted motion and sound energy from the video. We calculated motion energy as the pixel-wise change in successive frames of the gray scale version of the dance video, using a custom made matlab script^43^. The spectral power of the video soundtrack was computed using the miraudio toolbox for MATLAB^44^.

#### Summative audience engagement

After completion of the scanning and the follow-up viewing sessions, all participants completed a short quantitative performance engagement scale, see supplementary materials. Eight questions, addressed the participant’s level of engagement, i.e. “I was absorbed by what was happening in the performance”, “I found the performance interesting” or “I didn’t enjoy the performance”. A further four questions probed the participants felt understanding, i.e. “I felt like I understood what the performance was about” or “I couldn’t figure out why the performers were moving in particular ways” of the performance. One additional question asked participants to what extent they were distracted by scanner noise. All of 13 questions were answered on a 5-point Likert Scale ranging from Strongly Agree to Strongly Disagree. 7 questions were reverse coded.

#### Continuous audience engagement

To quantify each participant’s enjoyment of the performance outside of the scanner, ASUS touchscreen devices running the android version of OpenSesame experiment software were used^45^. A custom finger tracking experiment designed for investigation of live audience response continuously tracked the location of the participant’s index finger on the touch screen (24 Hz sampling rate). Shown on the touch screen was a single vertical axis (white text on black background), ranging from “Enjoy Very Much” at the top of the screen to “Enjoy Very Little” at the bottom of the screen. Participants were instructed to hold their right index finger on the device and move, continuously throughout the performance, in the direction most consistent with their current evaluation. For example, if the participant thought the performance was enjoyable, they should move their finger upwards. A practice session with the tablet was given before the performance began to allow participants to familiarize themselves with the tablet.

### Procedure

Scanning took place at the Combined Universities Brain Imaging Centre (CUBIC; www.cubic.rhul.ac.uk). On arrival, participants were given an overview of the imaging procedure, completed a short demographic questionnaire including dance expertise and were taken through a standard imaging safety report. Once participants were cleared to take part in MRI scanning, participants were asked to view a contemporary dance performance without music and were told that they would be asked to provide feedback on the performance after the scanning session.

Participants returned between two and three weeks after their fMRI scan, to complete a follow-up session where we collected the continuous enjoyment ratings. We chose to not collect continuous ratings during the fMRI session to better approximate the live viewing situation. Moreover, we requested a minimum of two weeks between fMRI and follow-up sessions in order to reduce the potential influence of familiarity with the video on continuous ratings. During the follow-up session, participants wore noise cancelling headphones and sat in front of a laptop to observe the same dance video as observed during the scanning session whilst making continuous enjoyment ratings on the tablet. At the completion of this follow-up session, participants were debriefed as to the purpose of the study.

### Behavioural data analyses

#### Summative audience engagement

To assess whether the experience of watching the performance was comparable between the first and the second viewing of the performance, we computed mean scores for engagement and understanding for each participant and submitted these to two 2 × 2 mixed ANOVAs with the factors session (FMRI vs. follow-up) and group (novices vs. dancers). We used a between subject t-test to see if there were any group differences in how much participants were distracted by scanner noise.

#### cross-recurrence analysis of continuous enjoyment ratings

Cross recurrence analysis is well suited to the dynamics of human behaviour^46^. Similarly to our analysis of movement synchrony among performers, we applied cross recurrence analysis to compute agreement of continuous enjoyment ratings among all spectators using the CRQA package in R^47^. We calculated the recurrence rate between pairs of continuous ratings, which quantifies their degree of temporal coupling. To assess how this temporal coupling changed over time, we used a windowed recurrence analysis, calculating pairwise recurrence rate every 3 s. These pairwise metrics can then be averaged over different groups. We computed two agreement measures. To test whether agreement differed between groups (H1) we computed pairwise cross recurrence between all expert dancers’ enjoyment ratings (dancers with dancers, hereafter DD) and all dance novices (novices with novices, hereafter NN) separately. Finally, to assess similarity between dancers, novices and the choreographer at the behavioural level, we computed Pearson correlations between the choreographer’s continuous enjoyment ratings and the average ratings of all dancers and all novices separately.

### fMRI data analyses

#### Data collection

MRI data were recorded with a Siemens 3 T MRI scanner (Magnetom Trio, Siemens, Erlangen, Germany) using a standard Siemens eight-channel array headcoil. Whole-brain functional images were acquired with a T2*-weighted gradient echo, echoplanar sequence sensitive to BOLD contrast (TR 3000 ms, TE 31 ms, voxel size 3 × 3x3mm) comprising 41 axial slices and a 192 mm FOV. During the experimental run, 680 volumes were acquired followed by a standard T1-weighted high-resolution (1 × 1 × 1 mm) anatomical scan of the head.

#### Pre-processing

All functional data was pre-processed using the FSL software library (Smith et al., 2004) in preparation for analysis with the ISC toolbox (see below). The 680-volume sequence was first motion corrected using MCFLIRT^48^ and the resulting series skull-stripped using BET^49^. A high-pass temporal filter was then applied with a cut-off of 60 s. All data were subsequently aligned to the MNI-152 2 mm template space in a multi-stage process using FLIRT^48^: first, the timeseries mean volume was aligned with the skull-stripped anatomical volume using a six-parameter rigid-body transformation; second, the anatomical volume was aligned with the MNI-152 template using a twelve-parameter affine transformation; third, the combined transforms were applied to the 4D timeseries data. Finally, the normalised functional data were smoothed with a Gaussian kernel with FWHM of 5 mm.

### ROIs analysis

In addition to whole brain analyses, we identified three regions of interest to disentangle three levels of processing of the dance video. These regions were selected based on group differences between dancers and novices and the extant literature. The first ROI covers two brain areas involved in audio-visual processing of dance videos, the superior temporal gyrus, BA41 and the medial occipital gyrus, BA 18^23^. The second area covers visual, motor and parietal brain areas involved in action observation^50^. These include the middle temporal gyrus (BA37), middle frontal gyrus (BA6), the inferior and superior parietal lobe (BA40/BA7). The third ROI includes brain areas of the default mode network which has previously been shown to also be involved in aesthetic judgement^15,16^. These areas include the prefrontal and orbitofrontal cortex (BA10/BA 47) as well as anterior and posterior cingulate cortices (BA32/BA31). ROIs were generated on the basis of Brodmann areas as defined in WFU Pickatlas^51^.

#### Inter-subject correlations (ISCs)

The data were analysed using the ISC toolbox version 3.0^52^. The voxel-wise ISC value represents the mean correlation coefficient calculated across all possible subject pairs^53^. A non-parametric bootstrapping method is used to test the significance of each ISC value, with each time series shifted by a random amount and the statistic recalculated 1 million times. Critical significance thresholds are then calculated by correcting *p*-values of the true realizations for each voxel using the false discovery rate (FDR) based multiple comparisons correction^54^. In parallel to the analysis of behavioural data we computed summative and continuous measures of ISCs.

#### Comparing ISCs between dancer and novices (H1)

To measure summative ISCs across the entire performance, we calculated pairwise ISCs for the whole time series (860 volumes), separately for dancers and novices.

#### Comparing ISCs between dancers, novices and the choreographer (H2)

To assess brain synchrony between the two groups of spectators and the choreographer, we computed pairwise ISCs between the choreographer and each dancer, and pairwise ISCs between the choreographer and each novice. A Fisher *z*-transform was applied to each correlation map followed by a 3 mm spatial smoothing filter. These were then used to test whether ISCs were significant between pairs by conducting voxel-wise non-parametric permutation tests (FSL Randomise^55^) utilising threshold-free cluster enhancement (TFCE) to correct for multiple comparisons and a fully exhaustive set of permutations of the data. Specifically, we conducted nonparametric 1-sample t-tests to establish areas of significant ISC between the choreographer and dancers, and between the choreographer and novices. We also conducted a nonparametric 2-sample unpaired t-test to establish regions of significant differences between choreographer/dancers and choreographer/novices ISC maps.

#### Predicting ISCs from performed movement synchrony (H3)

We used the wrist sensor and video data collected during the initial live performance experiment^21^ to link dynamic changes in spectators’ ISCs to the performers’ movements. We computed dynamic ISC maps for consecutive 30 s windows (10 volumes per window), across the length of the performance video, separated by 3 s (1 TR). This resulted in 671 ISC maps for the entire performance. The group-averaged mean time-series of the ten performers’ movements (synchrony and acceleration) and the video (motion energy and sound) were resampled to represent the same 30 s windows as the ISC and used as explanatory variables to predict temporal variation of ISCs within each voxel using a GLM approach.

All fMRI data analyses were carried out using FEAT (fMRI Expert Analysis Tool) Version 6.00, part of FSL (fMRIB’s Software Library, www.fmrib.ox.ac.uk/fsl). The only pre-statistics processing applied to the dynamic ISC data was spatial smoothing using a Gaussian kernel of FWHM 5 mm; high-pass temporal filtering was applied at the standard setting of 100 s. All explanatory variables were scaled to the interval [0,1] and convolved with a Gaussian filter (sigma 2.8 s, lag 5 s) and orthogonalized with respect to each other. The resulting *z*-statistic images were thresholded using clusters determined by *z* > 3.1 and a corrected cluster significance threshold of *p* < 0.05. These maps thus reflect the degree to which dynamic ISCs depend on the performers movements as measured directly during the live performance (synchrony and acceleration) or extracted from the video (motion and sound energy). Our fMRI dataset is available at https://openneuro.org/datasets/ds004783.

## Results

The results section is structured according to our three hypotheses. Firstly, we report summative and continuous enjoyment ratings and compare average ISCs between dancers and novices (H1). Secondly, we report summative ISCs between both groups and the choreographer as an implicit measure of artistic perspective sharing (H2). Thirdly, we assess to what extent dynamic ISCs in both groups are explained by exogenous features of the performance and endogenous aesthetic experience as measured by the similarity of continuous ratings (H3).

### Summative engagement and understanding

Ten novices and thirteen dancers completed both questionnaires immediately after the scanning session and following the second viewing of the dance video in the follow-up session, 2–3 weeks after rating their overall engagement with the performance video as whole.

Overall engagement with the dance video tended to be higher in dancers than in novices (*F*(1,21) = 3.3, *p* = 0.08, *η*^*2*^ = 0.14, mean novices = 3.16 (SE = 0.1), mean dancers = 3.48(SE = 0.11)). Engagement did not differ between fMRI and follow-up session and *F*(1,21) = 0.006, *p* = 0.93) and there was no interaction between session and group *F*(1,21) = 1.2, *p* = 0.28).

Dancers reported greater overall understanding of the dance video (*F*(1,21) = 9.4, *p* = 0.006, *η*^*2*^ = 0.31, mean novices = 2.46 (SE = 0.2), mean dancers = 3.29 (SE = 0.18)). Felt understanding did not differ between fMRI and follow-up sessions *F*(1,21) = 0.01, *p* = 0.85) and there was no interaction between session and group *F*(1,21) = 0.002, *p* = 0.97). Perceived distraction from noise did not differ between groups or sessions (all *p* = n. s.)

### Continuous enjoyment ratings (H1)

**Figure 2:**
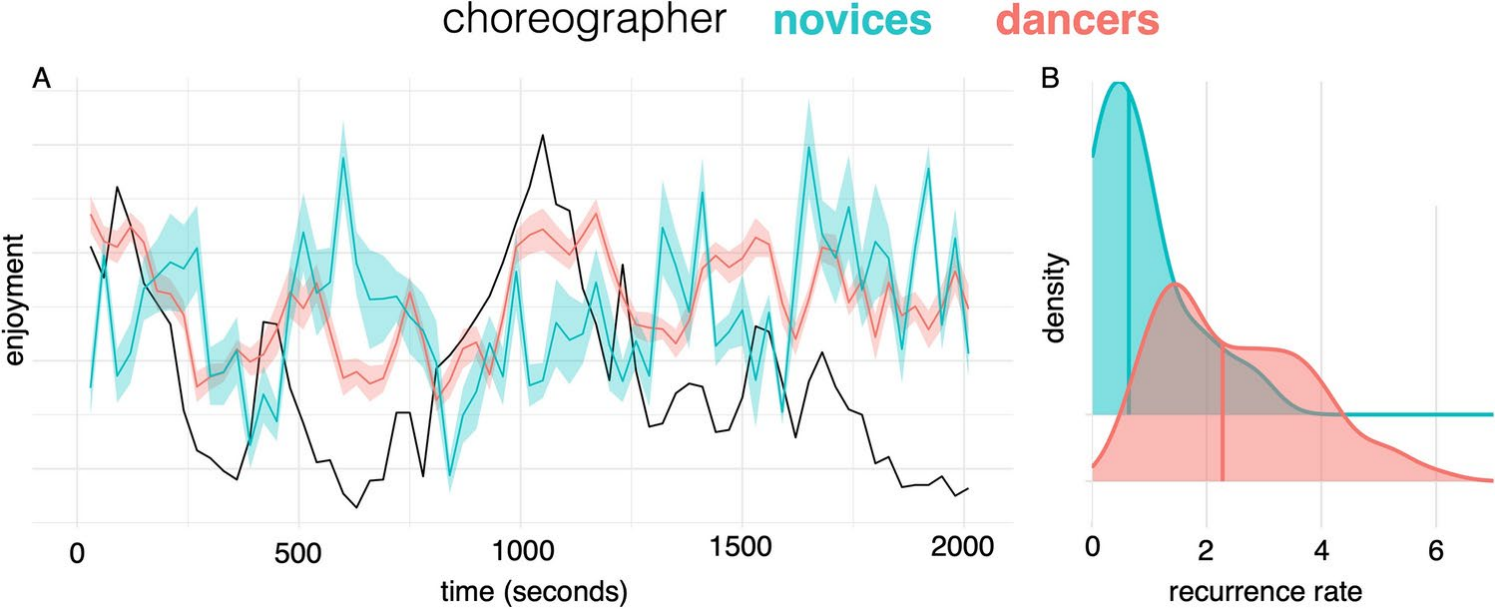
(**A**) Enjoyment ratings of the choreographer, and means of novices and dancers, with SEs shaded areas. (**B**) Temporal coupling time series are quantified in density plots of the recurrence rate between all dancer-dancer and novice-novice pairs.

Continuous enjoyment ratings were more similar between dancers than they were between dance novices. Figure 2A shows the ratings for the choreographer and the means of individual participants who are novices and dancers unfolding over time. Whereas the dancers’ mean ratings were correlated positively with the choreographer (*r* = 0.57, *p* < 0.0001), novices’ ratings were negatively correlated (*r* = *− *0.35, *p* < 0.005). There was also higher consistency within the dancers’ ratings than amongst the novices. The higher temporal coupling of dancers’ was shown by a windowed CRQA analysis. Figure 2B shows the distribution of mean recurrence rates, averaged across all time windows, for pairwise comparisons between dancers and other dancers, and novices and other novices. A t-test on these recurrence values showed a significant difference between the groups (*T*(30.2) = 6.61, *p* < 0.00001).

#### Average ISCs dancers vs. novices (H1)

**Fig. 3:**
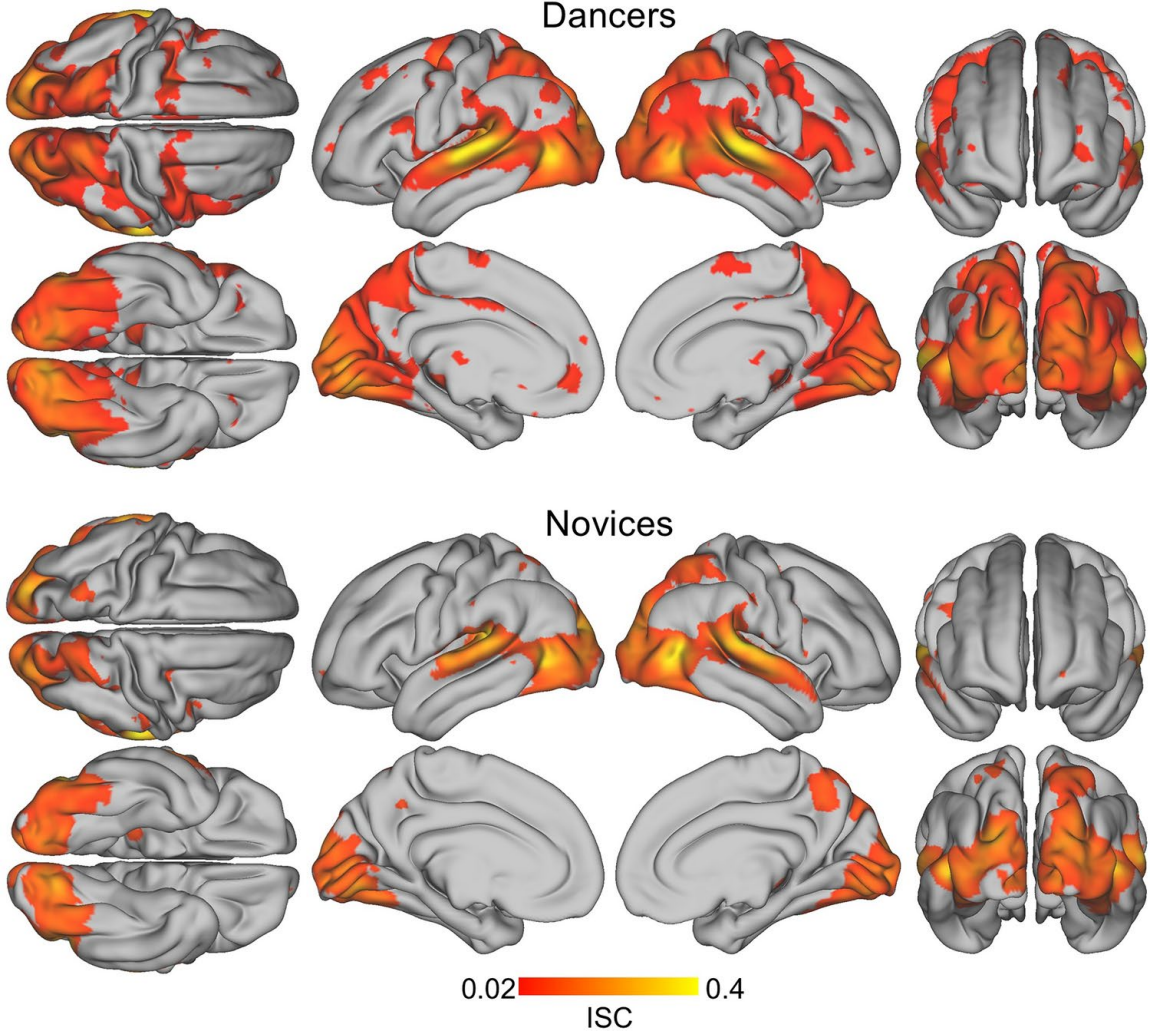
Group level ISC maps for dancers (top) and novices (bottom). These show statistically significant ISCs for each group (*p* < 0.001, FDR corrected) during viewing of the dance video.

We computed summative ISCs separately for Dancers and Novices. In both groups, we observe a large cluster encompassing bilateral areas of temporal, frontal, parietal and occipital cortices with peak activations in the superior and middle temporal gyri (see Fig. 3, Table 1). This cluster is nearly twice as large in dancers as in novices and includes subpeaks that in novices are either absent or separate from the main cluster. Notably, only dancers’ brains exhibit bilateral activations along the superior and inferior parts of the precentral gyrus including premotor and motor areas. Dancers also show stronger and more widespread similarity of activations in the middle occipital and lingual gyri (MOG) and the precuneus. Finally, dancers reveal significantly greater similarity of brain activations in the anterior (ACC) and posterior cingulate cortices (PCC), the angular gyrus, and the ventromedial prefrontal (vmPFC). A full table of all local maxima is included in the supplementary materials (S1). A direct comparison of the strength of ISCs between Dancers and Novices yielded significant differences in the superior temporal gyrus, bilaterally, the right middle temporal gyrus, and across inferior, middle and superior frontal gyri. However, only the bilateral activations in the STG survived FDR correction (see supplementary materials, S2).

**Table 1:** Cluster locations of the summative ISCs for Dancers and Novices.

Cluster region	Cluster extent (# voxels)	MNI coordinates (mm)			Peak statistic (*r*)
		x	y	z	
*Dancers*					
Superior temporal gyrus	69012^*a*^	60	− 28	8	0.3198
Anterior cingulate	349^*a*^	− 12	40	− 8	0.0290
Superior frontal gyrus	161	− 26	56	6	0.0273
Superior frontal gyrus	99	− 18	32	50	0.0260
Frontal pole	84^*a*^	24	68	0	0.0231
Anterior cingulate	66	12	50	− 8	0.0221
Inferior frontal gyrus	43	32	34	− 14	0.0225
Caudate	29	18	12	18	0.0203
Inferior frontal gyrus	28	− 26	28	− 12	0.0215
Medial frontal gyrus	22	− 2	56	20	0.0183
Superior frontal gyrus	18	28	50	12	0.0188
Middle frontal gyrus	16	− 34	16	54	0.0203
Postcentral gyrus	15	68	− 18	34	0.0233
Insula	14	36	8	8	0.0216
Cingulate gyrus	12	− 8	26	24	0.0196
*Novices*					
Superior temporal gyrus	28328^*a*^	50	− 18	4	0.2035
Precentral gyrus	237	48	4	30	0.0489
Middle frontal gyrus	230	22	− 4	46	0.0489
Superior parietal lobe	215	− 18	− 60	64	0.0444
Thalamus	108	16	− 28	− 6	0.0432
Cingulate gyrus	27	− 12	− 24	40	0.0330
Superior frontal gyrus	19	− 14	64	4	0.0295

Clusters were defined using the FSL *cluster* tool. ISC maps were thresholded at *p* < 0.001 (FDR corrected) and a cluster extent threshold of 96 mm^3^ applied.

^*a*^Local maxima were found for these clusters using a 20 mm separation threshold (see supplementary Table S1).

In sum, ISCs among dancers were overall more widespread, stronger and bilaterally symmetrical than those for the novices. In particular, ISCs along the precentral and superior, middle and inferior frontal gyri were only present for dancers but not for novices.

### Average ISCs with choreographer (H2)

**Fig. 4:**
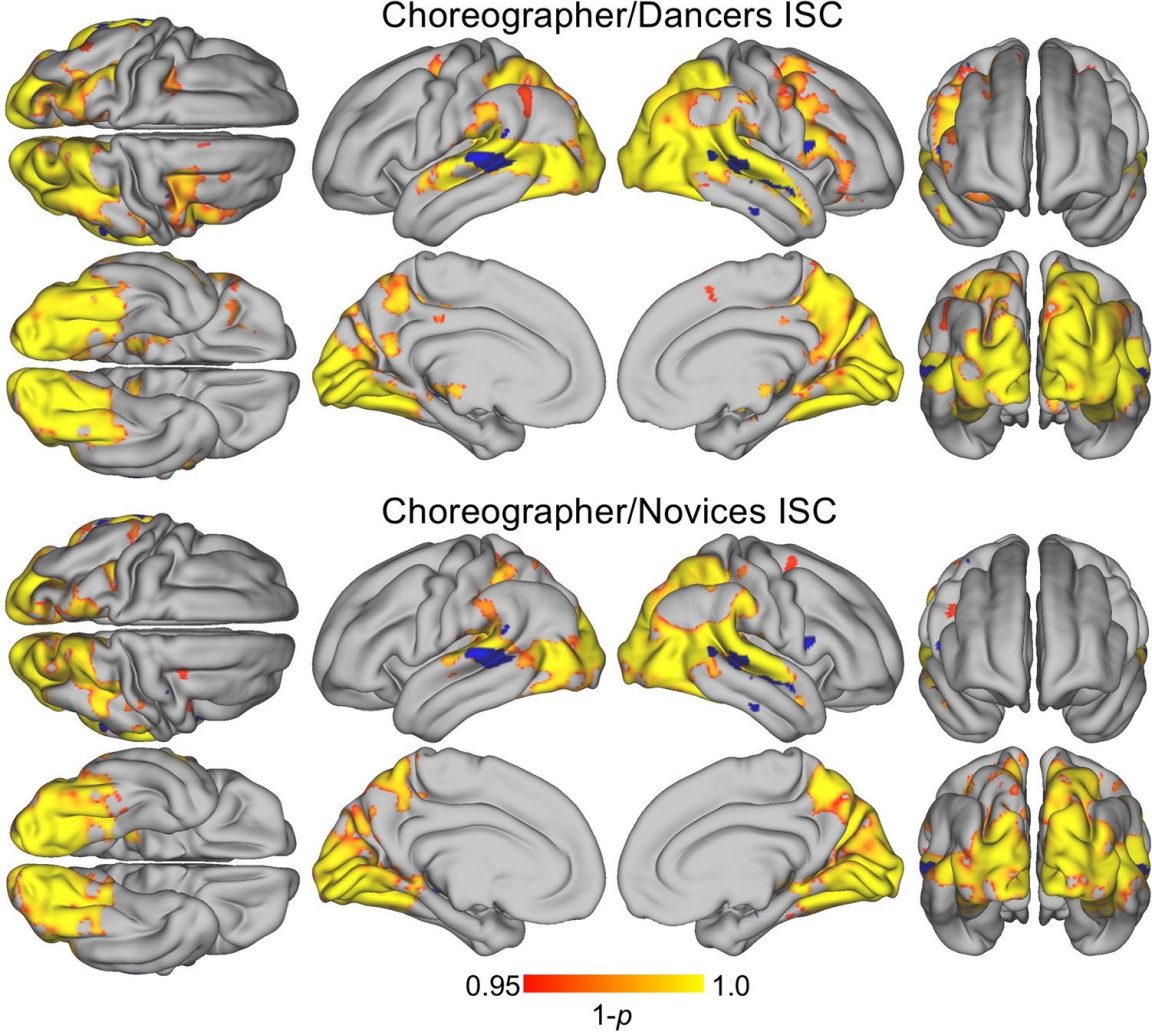
Regions of significant ISCs between choreographer and dancers (top) and choreographer and novices (bottom). Clusters derived using TFCE and a threshold of *p* < 0.05, FWE corrected. The blue clusters represent areas where ISCs display significant differences between the groups (dancers > novices, *p* < 0.01 uncorrected).

We computed ISCs between both groups of spectators and the choreographer as an indirect measure of sharing the choreographer’s perspective when viewing the dance video (Fig. 4, Table 2)^3,18,37^.

**Table 2:** Cluster locations of the summative ISCs for Dancers and Novices with the Choreographer.

Cluster region	Cluster extent (# voxels)	MNI coordinates (mm)			*p*-value
		x	y	z	
*Dancers and choreographer*					
Lateral occipital cortex	6612^*a*^	− 42	− 72	0	< 0.001
Superior temporal gyrus	5483^*a*^	62	− 10	− 2	< 0.001
Superior temporal gyrus	2442^*a*^	− 50	− 40	10	< 0.001
Inferior parietal lobule	836^*a*^	38	− 40	54	< 0.001
Inferior frontal gyrus	273^*a*^	38	12	20	0.006
Inferior parietal lobule	142	− 34	− 42	54	0.004
Precentral gyrus	76	− 34	− 8	50	0.005
Middle frontal gyrus	68	28	− 4	46	0.003
Superior occipital gyrus	35	36	− 72	34	0.015
Precuneus	20	22	− 66	26	0.028
Posterior cingulate gyrus	13	− 10	− 22	38	0.006
*Novices and choreographer*					
Occipital fusiform gyrus	2890^*a*^	− 18	− 74	− 16	< 0.001
Superior temporal gyrus	2691^*a*^	46	− 30	6	< 0.001
Superior temporal gyrus	939^*a*^	− 42	− 22	− 2	< 0.001
Lateral occipital cortex	133	− 48	− 68	− 2	0.003
Precuneus	86	4	− 60	54	0.011
Middle frontal gyrus	29	24	− 6	46	0.01
Lateral occipital cortex	22	− 48	− 72	− 16	0.01
*Dancers and choreographer > novices and choreographer (p < 0.001 uncorrected)*					
Superior temporal gyrus	204^*a*^	− 66	− 22	6	< 0.001
Superior temporal gyrus	194^*a*^	44	− 36	− 2	< 0.001
Inferior frontal gyrus	56	68	− 48	2	< 0.001
Precuneus	41	50	14	8	< 0.001
Cerebellum	19	48	− 72	40	< 0.001
Inferior temporal gyrus	16	− 34	− 80	− 50	< 0.001
Fusiform	12	60	− 18	− 28	< 0.001

Clusters were defined using the FSL *cluster* tool. ISC maps were thresholded at *p* < 0.05 (FWE corrected) and a cluster extent threshold of 96 mm^3^ applied.

^*a*^Local maxima were found for these clusters using a 20 mm separation threshold (see supplementary results, S3).

The pattern of shared brain activations for both dancers and novices with the choreographer resembles that of the basic ISC analyses for dancers and novices. For both groups, ISCs with the choreographer are strongest across right the superior temporal and middle temporal gyri. Smaller clusters of activation are located bilaterally in the lingual gyri, the right cuneus, and the right superior parietal sulcus. The pattern of activations is similar between dancers and novices, but ISCs between dancers and the choreographer are consistently larger than between novices and the choreographer. In case of the frontal activations, dancers show bilateral activations of motor cortices, whereas in novices these activations are typically limited to the right hemisphere. A direct comparison between dancers and novices, reveals significantly greater similarity (*p* < 0.001, uncorrected) bilaterally in STG and MTG, the right inferior frontal gyrus, the right fusiform gyrus and the precuneus, but these activations do not survive FDR correction. A full table of subpeaks is available in the supplementary material, S3).

### Dynamic ISCs in relation to performance features and continuous ratings (H3)

**Fig. 5:**
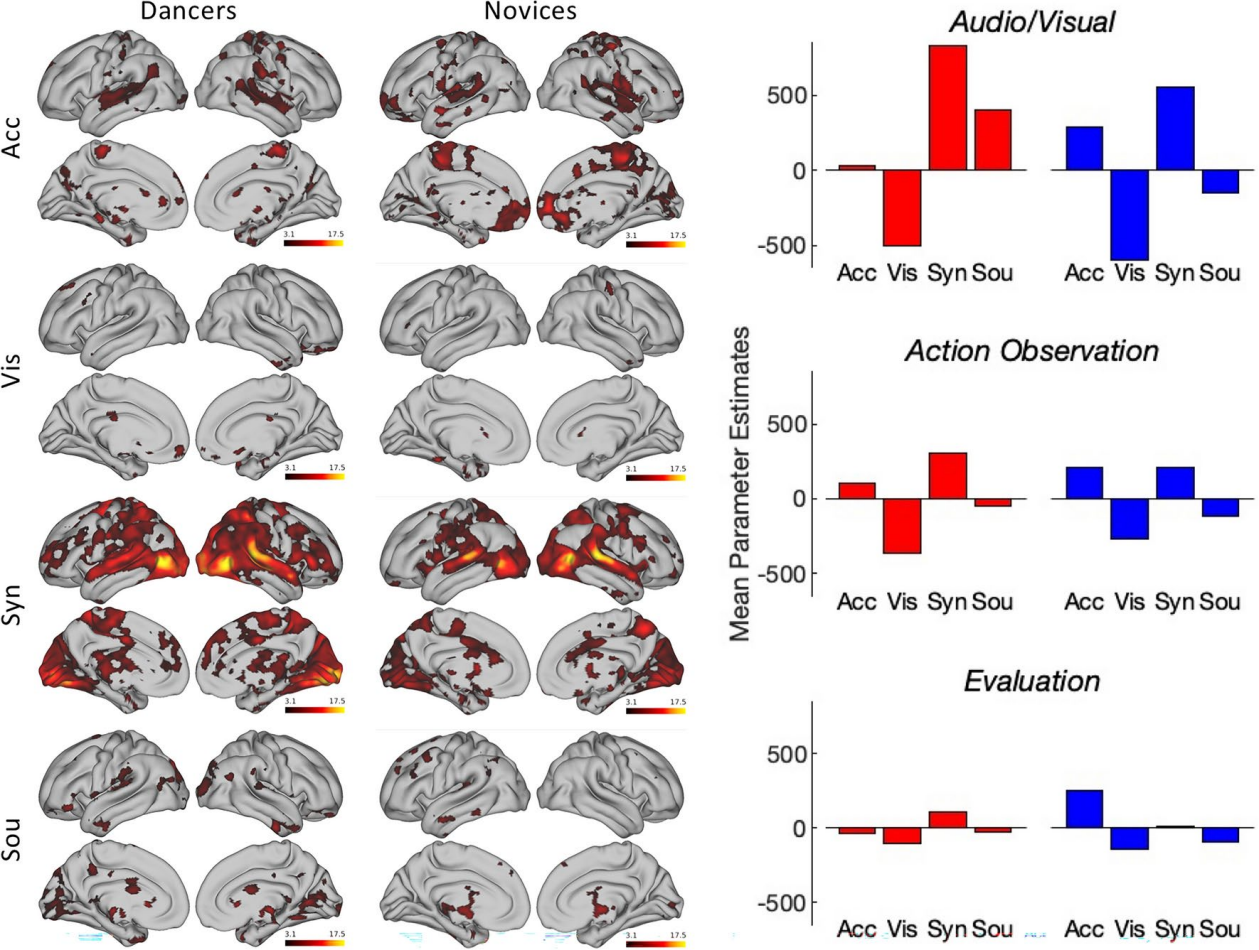
Relationship between dynamic ISCs and performance features. Left: cortical regions where ISCs are significantly associated with different performance features (Acc, performer acceleration; Vis, video motion; Syn, performer synchrony; Sou, video sound). *Z*-statistic images were thresholded using clusters determined by *z* > 3.1 and a corrected cluster significance threshold of *p* < 0.05. Right: Mean parameter estimates derived from three ROIs for the four performance features (red bars, dancers; blue bars, novices). See supplementary material for individual BAs.

We computed a regression analysis of dynamic ISCs and the average time series of both the performers movements, (movement acceleration and synchrony) and features of the videos (motion and sound energy), see Fig. 5. For both groups, synchrony emerged as the best predictor of ISCs, explaining approximately 10% of changes in ISCs. Accordingly, brain synchrony among both dancers and novices was most pronounced if dance performers moved in synchrony. Whereas ISCs among dancers were primarily related to synchrony only, ISCs among novices were better explained by a combination of synchrony and movement acceleration. This is particularly true for the brain areas encompassing the audiovisual ROI including the bilateral superior temporal gyri and the lingual and middle occipital gyri. Among novices, movement acceleration was a stronger predictor of ISCs in the evaluation ROI, including the ventromedial prefrontal cortex and the posterior and anterior cingulate cortices. In dancers, synchrony was a better predictor of ISCs overall, with stronger relationships across all three ROIs including in pre-motor and motor areas as well anterior and posterior cingulate cortices and dorsomedial prefrontal and orbitofrontal cortices (see supplementary materials for PEs for specific BAs). Both motion and sound energy were only weakly positive or negatively correlated with ISCs in both groups.

**Fig. 6:**
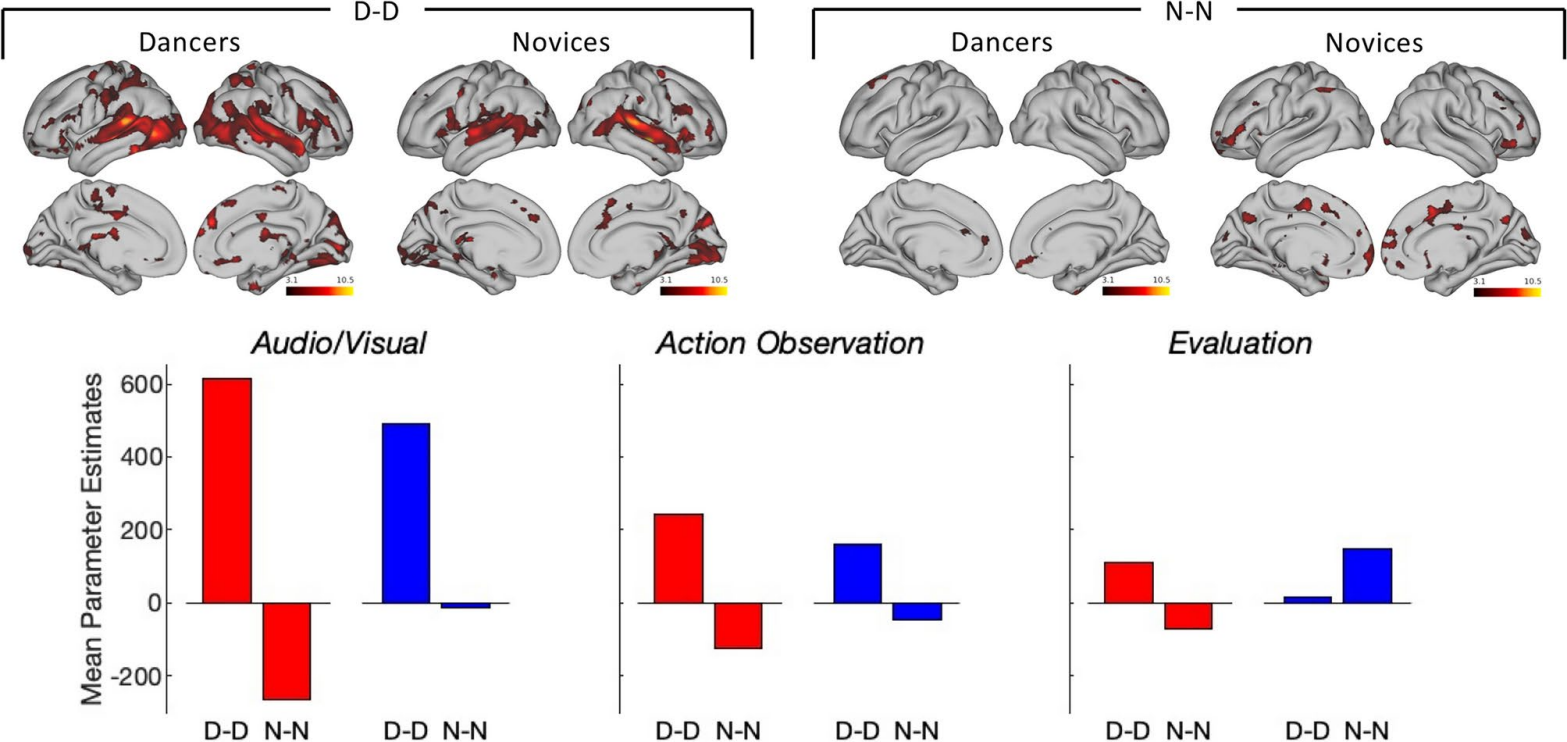
ISCs and CRQA of continuous enjoyment ratings. All performance measures were orthogonalized and convolved with a Gaussian function, spatial smoothing: 5 mm, T-contrasts, cluster threshold, z = 2.3, *p* < 0.05, FDR corrected. D-D refers to the similarity of enjoyment ratings among dancers, N–N refers to the similarity of enjoyment ratings among novices. See supplementary material for individual BAs.

Finally, we used windowed CRQA to compute the change over time of the similarity between groups’ continuous enjoyment ratings. We regressed this measure onto the similarity of brain activations within each group (see Fig. 6). For both audiovisual integration and action observation ROIs, similarity among dancers’ enjoyment was a better predictor of ISCs than similarity among the ratings of novices, particularly along the superior temporal gyrus and the middle temporal gyrus with two prominent subpeaks in these areas in both groups. In contrast, the ROI for stimulus evaluation revealed the anticipated pattern of group specificity, albeit with relatively small PEs: similarity of continuous enjoyment among dancers was a better predictor of similarity of brain active activations among dancers, whereas similarity of continuous enjoyment among novices was a better predictor of ISCs among novices. Group specificity was particularly pronounced in the anterior and posterior cingulate cortices (BA31, BA 32 and BA34) and the orbitofrontal cortex (BA10 and BA 47), see supplementary material for PEs for specific BAs.

## Discussion

Evolutionary biology argues that dance and music have evolved as social signalling systems, facilitating nonverbal communication between groups^2,21,56^. To test this hypothesis, we conducted an fMRI experiment to study whether behavioural and neural synchrony among people *watching* dance can be linked to movement synchrony among people *performing* dance, and to what extent behavioural and brain synchrony engagement and felt understanding depend on expertise in dance.

At the behavioural level we show that the dancers’ continuous enjoyment ratings are more similar to each other than the novices’ enjoyment ratings. Dancers’ ratings are also more similar to those of the choreographer. The continuous enjoyment ratings therefore provide clear evidence for a common aesthetic experience among dancers and among dancers and the choreographer^38^. Greater similarity of continuous ratings among dancers is accompanied but higher ratings of felt understanding of the dance video. Arguably, a common experience among dancers relates to shared knowledge^36^, in this case the choreographic principles and movement practices of contemporary performing dance. This shared knowledge also helps dancers to better understand the artistic perspective of the choreographer^18^ even if the specific dance work has not been experienced before. Somewhat paradoxically, our results suggest that dance expertise reduces the subjective variability of the aesthetic experience of dance. More generally, our findings suggest that expertise with an art form can reduce otherwise large individual differences of aesthetic judgement^14,57^ and of brain activations related to aesthetic judgement^15,16^.

Our fMRI findings revealed similar expertise effects at the neural level and show that enjoyment ratings are mediated by distinct dynamic features of the dance video. Both dancers and novices show highest ISCs in brain areas associated with audio-visual integration and action observation along the STS and the extra-striate body area, consistent with prior research on dance observation using fMRI and ISCs^23–25^. However, two important group differences emerge from our analyses. Firstly, only dancers show consistent bilateral activations of premotor and motor cortices, suggesting greater sensorimotor familiarity with the observed movements^24,31,32,35,58^. Secondly, although synchrony is the strongest predictor of ISCs in both groups, ISCs among novices are more strongly driven by the overall amount of movement of the group of performers^59,60^. Arguably, dancers more consistently focus on the relationships between performers rather than how much they are dancing^21^. Importantly, visual change and sound extracted from the video were only weakly or negatively correlated with ISCs among dancers and novices, in line with findings using narrative stimuli in which ISCs are better explained by higher level narrative rather than basic sensory features of the video^11^. Unsurprisingly, kinematics of the dancers’ movements are better captured by the accelerometers (3D) worn during the performance than the offline motion analysis of the video (2D), and our findings support previous research showing that changes in movement speed and synchrony are a key component of dance aesthetics^30,61–64^.

Finally, combining behavioural and neural measures our study provides insights into the question whether brain synchrony is primarily driven by stimulus features or whether it reflects a common experience^65,66^. Behavioural synchrony among dancers was the best predictor of brain synchrony in brain areas for audio-visual integration and for action observation across both groups of participants. One way to explain this seemingly paradoxical finding is that the dancers’ ratings tracked the video more reliably than the novices’ ratings which were less consistent over time. Therefore, ISCs in posterior brain areas primarily processing the movements and sounds of the performers were best explained by the dancers’ ratings because their ratings are more reliable and more accurately track the movement features in the video. Only frontal brain areas typically associated with stimulus evaluation (pre-, orbitofrontal and cingulate cortices) exhibited a group-specific pattern, in keeping with greater sensitivity of orbitofrontal and medial frontal cortices to subjective experience and self-referential processing in the default mode network^12,15,16,25,67^.

There are several limitations to our study. Firstly, the sample size of individual groups is relatively small. However, the close correspondence between behavioural and neural measures and reliable peak activations across both groups strongly indicate that group differences in brain activations reflect meaningful differences in subjective experience. Secondly, we identified ROIs broadly according Brodmann areas rather than using a localiser approach, due to the already long duration of the experiment. Overall, analysing our fMRI data in this conservative way is likely to reduce any potential group differences rather than amplify them. Inter-subject correlations in the medial frontal cortex in particular are highly variable across individuals^12^, yet our between-subject design is able to identify meaningful relationships between aesthetic judgement and ISCs in these areas.

In sum, our findings provide strong evidence for a common experience between performers and spectators of a dance performance and—additionally—the choreographer of the dance performance in keeping with the importance of nonverbal communication for the aesthetic appreciation of dance^3,37^. Expertise with dance facilitates this common experience^36,38^ which—in the case of dance—involves not only visual but also motor areas of the brain. More generally, our findings support the idea that ISCs measured among individual viewers in the scanner indeed reflect a common experience rather than merely watching the same stimulus, as the strength of the ISCs depends on prior experience with the art form and—for the dancers—the ability to better share the choreographers’ artistic perspective when viewing the work.

## Supplementary Information

Supplementary Information.

## Data Availability

The data supporting this study are openly available at https://openneuro.org/datasets/ds004783. The dance video shown in the scanner is available at https://research.gold.ac.uk/id/eprint/34238/.
