# Supplementary material for: Movement synchrony among dance performers predicts brain synchrony among dance spectators
Source: Sci Rep. 2024 Sep 27;14:22079. doi: 10.1038/s41598-024-73438-0 (PMC11436841; doi:10.1038/s41598-024-73438-0)
Supplement: Supplementary file 1 — Supplementary Information. [file 41598_2024_73438_MOESM1_ESM.docx]

**Supplementary Materials**

*Table S1: Local Maxima ISCs Dancers and Novices determined using FSL “cluster” – H1.*

| Local Maxima Region | Cluster Index | MNI Coordinates (mm) | | | Local Maxima |
| --- | --- | --- | --- | --- | --- |
|  |  | x | y | z |  |
| **Dancers** | | | | | |
| Superior temporal gyrus | 1 | 60 | -28 | 8 | 0.32 |
| Superior temporal gyrus | 1 | -46 | -26 | 4 | 0.298 |
| Superior temporal gyrus | 1 | 40 | -30 | 12 | 0.232 |
| Middle temporal gyrus | 1 | 48 | -66 | 2 | 0.219 |
| Middle temporal gyrus | 1 | -46 | -72 | 2 | 0.208 |
| Lingual gyrus | 1 | 10 | -78 | -6 | 0.159 |
| Lingual gyrus | 1 | -8 | -84 | 4 | 0.145 |
| Middle occipital gyrus | 1 | -22 | -90 | 20 | 0.145 |
| Middle occipital gyrus | 1 | 24 | -86 | 22 | 0.132 |
| Lingual gyrus | 1 | 14 | -96 | 6 | 0.13 |
| Fusiform gyrus | 1 | -22 | -74 | -10 | 0.114 |
| Superior parietal lobe | 1 | 18 | -60 | 60 | 0.106 |
| Fusiform gyrus | 1 | 42 | -50 | -16 | 0.0932 |
| Superior parietal lobe | 1 | 36 | -40 | 54 | 0.0852 |
| Superior parietal lobe | 1 | -20 | -56 | 60 | 0.0844 |
| Middle frontal gyrus | 1 | -20 | -8 | 58 | 0.0767 |
| Superior parietal lobe | 1 | -32 | -38 | 46 | 0.0749 |
| Precentral Gyrus | 1 | -40 | -8 | 50 | 0.0707 |
| Cerebellum | 1 | -38 | -60 | -20 | 0.0706 |
| Posterior Cingulate Gyrus | 1 | -12 | -26 | 38 | 0.068 |
| Precuneus | 1 | 4 | -54 | 46 | 0.0662 |
| Fusiform Gyrus | 1 | 34 | -74 | -16 | 0.0621 |
| Cuneus | 1 | 8 | -86 | 42 | 0.0571 |
| Fusiform Gyrus | 1 | -20 | -52 | -10 | 0.055 |
| Thalamus | 1 | 12 | -28 | -6 | 0.0534 |
| Precentral gyrus | 1 | 30 | -4 | 50 | 0.0518 |
| Middle frontal gyrus | 1 | 54 | 22 | 4 | 0.0517 |
| Parahippocampal gyrus | 1 | 30 | -50 | 4 | 0.049 |
| Precuneus | 1 | -22 | -70 | 38 | 0.047 |
| Thalamus | 1 | -14 | -30 | -4 | 0.0466 |
| Cerebellum | 1 | -8 | -78 | -40 | 0.0407 |
| Inferior Frontal Gyrus | 1 | 50 | 12 | 22 | 0.0403 |
| Precentral Gyrus | 1 | 54 | -2 | 40 | 0.04 |
| Angular Gyrus | 1 | 56 | -58 | 20 | 0.0378 |
| Supramarginal gyrus | 1 | 54 | -26 | 38 | 0.0361 |
| Posterior cingulate gyrus | 1 | 12 | -24 | 42 | 0.0345 |
| Planum Polare | 1 | 42 | -8 | -12 | 0.0304 |
| Posterior cingulate gyrus | 1 | -18 | -44 | 14 | 0.0303 |
| Postcentral Gyrus | 1 | -52 | -24 | 36 | 0.0296 |
| Inferior frontal gyrus | 1 | -46 | 12 | 20 | 0.0295 |
| Superior frontal gyrus | 1 | 8 | 4 | 66 | 0.029 |
| Inferior parietal lobe | 1 | 50 | -62 | 46 | 0.0282 |
| Middle frontal gyrus | 1 | 48 | 38 | 20 | 0.0265 |
| Posterior cingulate gyrus | 1 | -18 | -64 | 10 | 0.0245 |
| Culmen | 1 | -28 | -34 | -28 | 0.0244 |
| Anterior cingulate gyrus | 1 | -4 | 6 | 36 | 0.0244 |
| Angular gyrus | 1 | -40 | -56 | 32 | 0.024 |
| Posterior cingulate gyrus | 1 | 2 | -54 | 8 | 0.0238 |
| Superior frontal gyrus | 1 | -6 | -10 | 76 | 0.0237 |
| Posterior cingulate gyrus | 1 | 22 | -64 | 18 | 0.0234 |
| Anterior cingulate gyrus | 2 | -12 | 40 | -8 | 0.029 |
| Medial frontal gyrus | 2 | -2 | 58 | -2 | 0.0197 |
| Frontal pole | 3 | 24 | 68 | 0 | 0.0231 |
| Frontal pole | 3 | 42 | 50 | 4 | 0.0186 |
| **Novices** | | | | | |
| Superior temporal gyrus | 1 | 50 | -18 | 4 | 0.203 |
| Superior temporal gyrus | 1 | -48 | -26 | 4 | 0.177 |
| Middle temporal gyrus | 1 | 48 | -68 | 2 | 0.162 |
| Middle temporal gyrus | 1 | -46 | -68 | 0 | 0.131 |
| Middle occipital gyrus | 1 | -22 | -90 | 14 | 0.114 |
| Middle occipital gyrus | 1 | 26 | -90 | 12 | 0.109 |
| Fusiform gyrus | 1 | -18 | -72 | -14 | 0.0974 |
| Insula | 1 | 40 | -34 | 16 | 0.0928 |
| Cuneus | 1 | 26 | -82 | 32 | 0.0884 |
| Lingual gyrus | 1 | 16 | -92 | -6 | 0.0834 |
| Fusiform gyrus | 1 | 24 | -68 | -16 | 0.074 |
| Lingual gyrus | 1 | -10 | -90 | -8 | 0.0731 |
| Superior temporal gyrus | 1 | 60 | 0 | -6 | 0.0703 |
| Fusiform gyrus | 1 | 44 | -54 | -18 | 0.0621 |
| Precuneus | 1 | 24 | -56 | 58 | 0.0621 |
| Precuneus | 1 | 4 | -56 | 46 | 0.0529 |
| Cerebellum | 1 | -42 | -64 | -22 | 0.0459 |
| Posterior cingulate gyrus | 1 | 34 | -66 | 20 | 0.044 |
| Middle temporal gyrus | 1 | 58 | -46 | 2 | 0.04 |
| Inferior parietal lobe | 1 | 58 | -26 | 34 | 0.0383 |
| Inferior parietal lobe | 1 | 40 | -38 | 54 | 0.0352 |
| Insula | 1 | -48 | -22 | 24 | 0.0344 |
| Superior temporal gyrus | 1 | 50 | 18 | -22 | 0.0328 |
| Superior temporal gyrus | 1 | -62 | 0 | -10 | 0.0322 |
| Middle temporal gyrus | 1 | -64 | -50 | 0 | 0.0282 |
| Culmen | 1 | -2 | -64 | 2 | 0.0279 |

*Note*. Minimum number of local maxima = 50, minimum distance apart = 20 mm, cluster extent threshold= 96 mm^3^

*Table S2:* *ISC difference maps cluster locations for Dancers>Novices – H1*

| Cluster Region | Cluster Extent (# voxels) | MNI Coordinates (mm) | | | Peak Statistic (*z*) |
| --- | --- | --- | --- | --- | --- |
|  |  | x | y | z |  |
| **Dancers > Novices (*p* < 0.05 FDR corrected)** | | | | | |
| Superior temporal gyrus | 491 | 58 | -34 | 4 | 5.41 |
| Superior temporal gyrus | 125 | -58 | -26 | 6 | 4.64 |
| **Dancers > Novices (*p* < 0.01 uncorrected)** | | | | | |
| Superior temporal gyrus | 2270 | 58 | -34 | 4 | 5.41 |
| Superior temporal gyrus | 1590 | -58 | -26 | 6 | 4.64 |
| Lingual gyrus | 953 | 10 | -76 | -8 | 3.10 |
| Occipital pole | 244 | -10 | -100 | 18 | 2.57 |
| Superior parietal lobe | 196 | 16 | -54 | 70 | 2.41 |
| Lateral occipital cortex | 135 | 34 | -86 | 30 | 2.63 |
| Superior parietal lobe | 102 | -32 | -40 | 46 | 2.71 |
| Lateral occipital cortex | 84 | -48 | -74 | 4 | 2.32 |
| Superior frontal gyrus | 60 | -24 | -4 | 62 | 2.37 |
| Superior parietal lobe | 51 | -24 | -54 | 60 | 1.90 |
| Occipital pole | 50 | 12 | -94 | 32 | 2.13 |
| Occipital pole | 43 | -20 | -92 | 34 | 2.30 |
| Insula | 33 | 56 | -38 | 22 | 2.56 |
| Fusiform gyrus | 32 | -40 | -52 | -14 | 2.33 |
| Precentral gyrus | 26 | -40 | -8 | 50 | 2.09 |
| Middle temporal gyrus | 23 | -46 | -58 | 0 | 2.12 |
| Fusiform gyrus | 19 | 42 | -48 | -16 | 2.15 |
| Superior frontal gyrus | 18 | 18 | -2 | 62 | 2.00 |
| Occipital pole | 17 | 14 | -96 | 6 | 1.93 |
| Insula | 17 | -46 | -40 | 20 | 2.28 |

*Note*. Clusters were defined using the FSL *cluster* tool. ISC maps were thresholded as indicated above and a cluster extent threshold of 96 mm^3^ applied. No clusters survived statistical thresholding for the opposite comparison, Novices > Dancers.

*Table S3: Local Maxima ISCs Dancers/Novices with choreographer determined using FSL “cluster” – H2.*

| Local Maxima Region | Cluster Index | MNI Coordinates (mm) | | | p-value |
| --- | --- | --- | --- | --- | --- |
|  |  | x | y | z |  |
| **Dancers and Choreographer** | | | | | |
| Middle temporal gyrus | 1 | -42 | -72 | 0 | <.001 |
| Occipital pole | 1 | -8 | -100 | 10 | <.001 |
| Lingual gyrus | 1 | -4 | -82 | -6 | <.001 |
| Fusiform gyrus | 1 | -22 | -74 | -12 | <.001 |
| Occipital pole | 1 | 14 | -94 | 0 | <.001 |
| Fusiform gyrus | 1 | -40 | -50 | -18 | <.001 |
| Fusiform gyrus | 1 | 26 | -64 | -10 | <.001 |
| Cuneus | 1 | 24 | -88 | 24 | <.001 |
| Inferior occipital gyrus | 1 | -30 | -94 | -2 | <.001 |
| Superior temporal gyrus | 2 | 62 | -10 | -2 | <.001 |
| Middle temporal gyrus | 2 | 64 | -30 | 2 | <.001 |
| Middle occipital gyrus | 2 | 52 | -70 | -6 | <.001 |
| Fusiform gyrus | 2 | 44 | -52 | -18 | <.001 |
| Insula | 2 | 50 | -36 | 22 | <.001 |
| Inferior occipital gyrus | 2 | 36 | -88 | -2 | <.001 |
| Middle temporal gyrus | 2 | 52 | 4 | -22 | <.001 |
| Superior temporal gyrus | 3 | -50 | -40 | 10 | <.001 |
| Insula | 3 | -44 | -22 | 0 | <.001 |
| Inferior Parietal Lobe | 4 | 38 | -40 | 54 | <.001 |
| Precuneus | 4 | 18 | -56 | 58 | <.001 |
| Inferior frontal gyrus | 5 | 38 | 12 | 20 | <.006 |
| Middle frontal gyrus | 5 | 40 | -2 | 44 | <.018 |
| **Novices and Choreographer** | | | | | |
| Lingual gyrus | 1 | -18 | -74 | -16 | <.001 |
| Lingual gyrus | 1 | 12 | -74 | -2 | <.001 |
| Middle occipital gyrus | 1 | -22 | -94 | 12 | <.001 |
| Middle occipital gyrus | 1 | 24 | -90 | 20 | <.006 |
| Superior temporal gyrus | 2 | 46 | -30 | 6 | <.001 |
| Middle temporal gyrus | 2 | 48 | -70 | 0 | <.001 |
| Superior temporal gyrus | 2 | 62 | -16 | 2 | <.001 |
| Insula | 3 | -42 | -22 | -2 | <.001 |
| Superior temporal gyrus | 3 | -50 | -38 | 10 | <.001 |
| Insula | 3 | -30 | -30 | 14 | <.001 |
| **Dancers and Choreographer > Novices and Choreographer** | | | | | |
| Superior temporal gyrus | 1 | -66 | -22 | 6 | <.001 |
| Middle temporal gyrus | 1 | -52 | -42 | 2 | <.001 |
| Superior temporal gyrus | 2 | 44 | -36 | -2 | <.001 |
| Middle temporal gyrus | 2 | 64 | -30 | -2 | <.001 |

*Note*. Minimum number of local maxima = 50, minimum distance apart = 20 mm, cluster extent threshold= 96 mm^3^

^
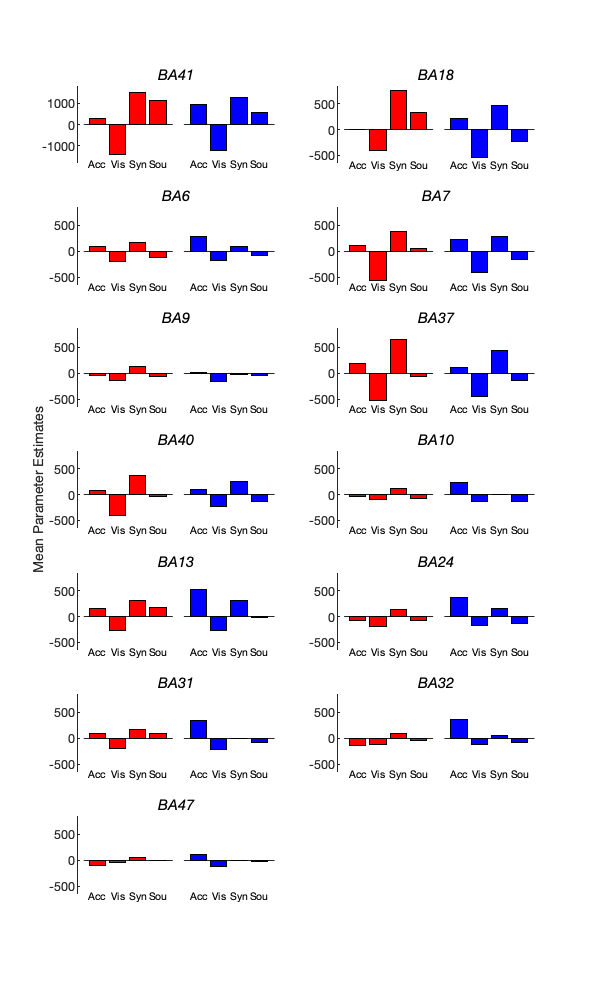
^

Figure S4: Parameter estimates performance features for individual Brodmann areas (H3)

^
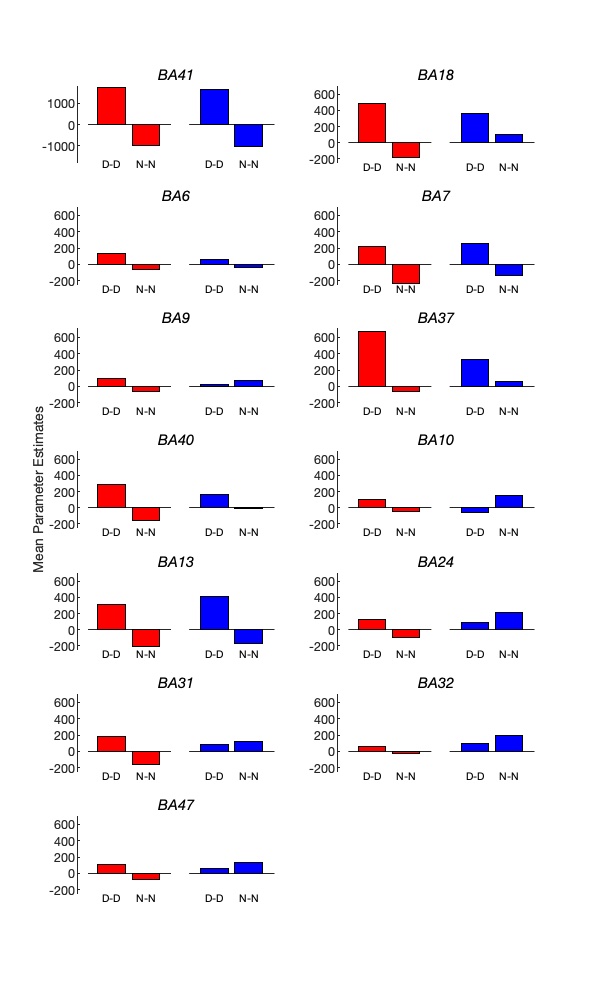
^

Figure S5: Parameter estimates for continuous enjoyment ratings for individual Brodmann areas (H3)

**Post Session Questionnaire**

**Aesthetic Preferences in Performance Art: An fMRI Study**

Please indicate the degree to which you agree or disagree with the statements below by putting an X in the appropriate box. Please read each statement carefully before making your decision.

|  |  | **Strongly Agree** | **Agree** | **Neither Agree or Disagree** | **Disagree** | **Strongly Disagree** |
| --- | --- | --- | --- | --- | --- | --- |
| E1 + | I was absorbed by what was happening in the performance |  |  |  |  |  |
| E2 - | I was easily distracted while watching the performance |  |  |  |  |  |
| E3 - | The performance didn’t really hold my attention |  |  |  |  |  |
| E4 + | I felt immersed in the sights and sounds of the performance |  |  |  |  |  |
| E5 - | I felt tired and uninterested |  |  |  |  |  |
| U1 - | I didn’t feel like I could identify with the performance |  |  |  |  |  |
| E6 + | I hardly noticed time passing during the session |  |  |  |  |  |
| U2 + | I felt like I understood what the performance was about |  |  |  |  |  |
| E7 - | I didn’t enjoy the performance |  |  |  |  |  |
| U3 + | I feel like I understood what the choreographer was trying to do in the performance |  |  |  |  |  |
| U4 - | I couldn’t figure out why the performers were moving in particular ways |  |  |  |  |  |
| Noise | I found it hard to concentrate because of noise |  |  |  |  |  |
| E8 + | I found the performance interesting |  |  |  |  |  |

S6: Post-scanning questionnaire assessing summative engagement and understanding. Overall engagement (E1 – E8) and understanding scores (U1 – U4) were obtained by averaging across individual items for each participant.
